# An Efficient Frequency Estimator for a Complex Exponential Signal Based on Interpolation of Selectable DTFT Samples

**DOI:** 10.3390/s22030861

**Published:** 2022-01-23

**Authors:** Miaomiao Wei, Aihua Zhang, Lin Qi, Bicao Li, Jun Sun

**Affiliations:** 1 Department of Electronic and Information, Zhongyuan University of Technology, Zhengzhou 450007, China; zhah@zut.edu.cn (A.Z.); 6524@zut.edu.cn (B.L.); 5391@zut.edu.cn (J.S.); 2 Department of Information Engineering, Zhengzhou University, Zhengzhou 450001, China; ielqi@zzu.edu.cn

**Keywords:** frequency estimation, spectral analysis, parameter estimation, DTFT interpolation

## Abstract

The frequency estimation of complex exponential carrier signals in noise is a critical problem in signal processing. To solve this problem, a new iterative frequency estimator is presented in this paper. By iteratively computing the interpolation of DTFT samples, the proposed algorithm obtains a fine frequency estimate. In addition, its mean square error (MSE) analysis is presented in this paper. By analyzing influences of the selectable parameters on the estimation accuracy of the model, a method for choosing appropriate parameters is discussed, helping to reduce the estimation error of the proposed estimator. Simulation results show that compared with other algorithms with a comparable estimation accuracy, the proposed iterative estimator can obtain a root mean square error (RMSE) that is closer to Cramér–Rao lower bound (CRLB).

## 1. Introduction

Obtaining accurate and fast frequency estimates of complex exponential signals is a classic problem in signal processing. It has extensive applications in navigation [1], power systems [2,3], metabolomics [4], and aerospace communication [5,6]. The frequency offsets in these systems may lead to demodulation failure, especially for highly dynamic applications, such as satellite communication, aviation communication control systems, and missile control systems. Thus, an efficient and simple frequency estimation method is necessary to ensure real-time communication for the above applications.

Frequency estimation can be seen as a search process. A coarse frequency estimate can be obtained by searching the spectral maximum. However, the estimation accuracy of algorithms based on the spectrum calculated by a DFT is limited by the frequency resolution. The obtained estimate must be an integral multiple of the frequency resolution, which deviates from the fact that the true frequency usually lies between spectral lines. Thus, it is necessary to determine a fine frequency estimation to further reduce the residual frequency offset.

Many estimators have been proposed based on the interpolation of DFT and DTFT samples, which is an efficient way to perform fine frequency estimation [7,8,9,10,11,12,13,14,15,16,17,18,19,20,21,22,23,24,25,26,27]. The interpolations of Jacobsen estimator [7], Candan estimator [8,9], Quinn estimator [10], parabolic interpolation estimator [11,12], and Fang estimator [13] are based on DFT samples. The residual frequency offset can be reduced with only a few additional multiplication operations after obtaining a coarse estimation. However, the RCSTL estimator [14], which uses three DTFT samples to assist with fine frequency estimation, obtains a smaller estimation error than other approaches. Its estimation error still deviates from the CRLB. Therefore, A&M in [16] first introduced the iteration operation into interpolation algorithms and further reduced the offset. IpDFT/e-IpDFT [19] were proposed to reduce the spectral leakage of coarse estimation by iteratively interpolating the weighted signal. The weighted signal is generated by applying different windows on the received signal [20,21,22,23]. Moreover, iterative algorithms can also obtain precise frequency estimates for various signals, such as the real sinusoid signal [26], the damped complex exponential signal [28], and the two-dimensional (2-D) complex exponential signal [29]. The asymptotic variances of some iterative estimators are very close to the CRLB, but an edge effect problem is encountered in which the variance may increase obviously at the edge of the frequency offset range. At the end, we summarize the performance of the key estimators in Table 1:

In fact, even a slight improvement in the estimation accuracy is important for signals in highly dynamic applications. Thus, to further explore the performance potential of estimators based on interpolation and obtain robust estimates, we present a new iterative algorithm in this paper. It includes two stages. In the coarse estimation stage, we first pad M−N zeros after the *N*-point signal samples. Then, a coarse estimate can be obtained by maximizing the magnitude of the *M*-point FFT, which is an efficient computation method of the DFT. In the fine estimation stage, every iteration, three DTFT samples are calculated to obtain a fine estimate. The general form of the estimator and a mean square error (MSE) analysis are also presented so that the estimator parameters can be adjusted according to the given design requirements. Simulation results reveal that the RMSE of the proposed estimator is closest to the CRLB in most cases. At the same time, this estimator has a relatively simple form and low computational complexity, so it can satisfy the high accuracy and fast estimation speed requirements of the given application environments. It can not only be used in a single complex signal system but can also be used for signal estimation in multi-sinusoid communication systems when introducing the approach proposed in [27].

The rest of the paper is organized as follows. In Section 2, the deduction of the model expression is presented, and the estimation process is also described. In Section 3, a general MSE analysis of the new estimator is performed. The developed parameter design method is discussed in Section 4. In Section 5, a comparison between the new estimator and existing estimators is given by simulation.

## 2. Signal and Frequency Offset Model

For the frequency estimation of complex exponential signals in noise, the received signal can be written as [11]

(1) $$\begin{array}{cc} y(n) & =x(n)+w(n)\\ \; & =Ae^{\left(j\left(2\pi \frac{f}{f_{s}}n+\phi \right)\right)}+w\left(n\right),n=0,1,\cdots ,N-1, \end{array}$$

where $x\left(n\right)=Ae^{\left(j\left(2\pi \frac{f}{f_{s}}n+\phi \right)\right)}$ is the useful signal, *A* is the signal magnitude, ϕ is the initial phase, $f_{s}$ is the sampling rate, *N* is the number of signal samples, and *f* is the signal frequency that needs to be estimated. w(n) represents complex Gaussian white noise following a normal distribution $N(0,\sigma^{2})$.

### 2.1. CRLB of Frequency Estimation

The Cramér–Rao Lower Bound of frequency estimation is [30]

(2) $$CRLB=\frac{3}{2\pi^{2}SNR\cdot N\left(N^{2}-1\right)},$$

where SNR is the signal-to-noise ratio (*SNR*). Its definition is $SNR=\frac{A^{2}}{\sigma^{2}}$.

### 2.2. DFT and DTFT of Complex Exponential Signals

First, the *M*-point DFT of x(n) can be obtained by

(3) $$X\left(k\right)=\sum_{n=0}^{M-1}x\left(n\right)e^{-j\frac{2\pi nk}{M}}.$$

It is assumed that *M*-point data are generated by padding an integral multiple of M−N zeros after *N* signal samples; for example, M=3N means that *N* signal samples are padded with 2N zeros. (3) can be written as

(4) $$X\left(k\right)=\sum_{n=0}^{N-1}Ae^{j\phi }e^{j2\pi \frac{f}{f_{s}}n}e^{-j\frac{2\pi nk}{M}}.$$

In (3), noise is not considered for simplifying the estimation process. The spectral line can be found by locating the maximum of $\left|X(k)\right|$ and is denoted as $k_{m}$, $k_{m}\in \left[1,M\right]$. The corresponding frequency estimation is $k_{m}\Delta f$. $\Delta f=f_{s}/M$ is the frequency resolution of the spectrum of the input complex exponential signal. Since the actual spectral peak usually lies between spectral lines $f=(k_{m}\pm \delta )\Delta f$, where δ∈[−0.5,0.5] is the residual frequency offset between the true frequency and the coarse frequency estimate $k_{m}\Delta f$, a smaller frequency resolution is helpful for obtaining a higher frequency estimation accuracy. Thus, frequency resolution has an important impact on the accuracy of frequency estimation. To reduce this influence, it is necessary to perform a second stage to refine the frequency estimation process. This is an efficient method for performing fine estimation using DTFT samples around the DFT peak $k_{m}$. DTFT samples are usually regarded as interpolations between DFT samples. The DTFT samples around $k_{m}$ can be expressed as

(5) $$X\left(k_{m}+p\right)=\sum_{n=0}^{N-1}Ae^{j\phi }e^{j2\pi \frac{f}{f_{s}}n}e^{-j\frac{2\pi nk}{M}}{|}_{k=k_{m}+p,f=\left(k_{m}+\delta \right)\Delta f}.$$

**Figure 1:**
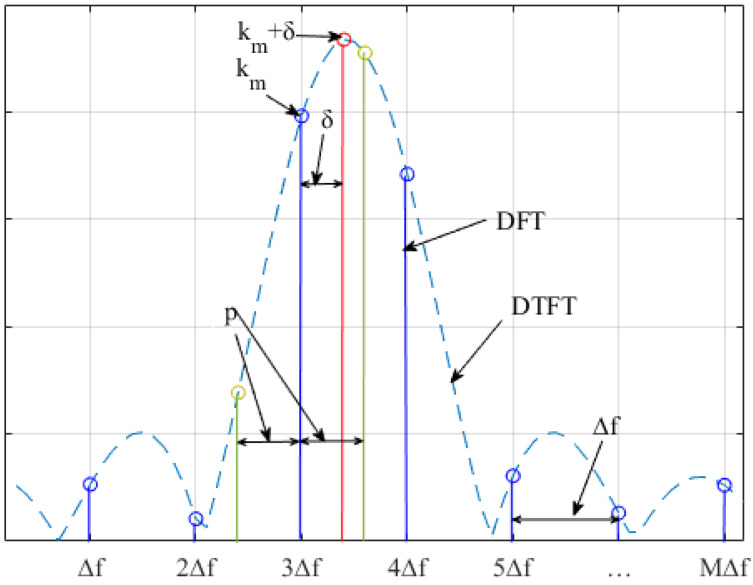
Magnitudes of the DTFT and DFT of a complex exponential signal.

As shown in Figure 1, the two neighboring green lines of $k_{m}$ are the DTFT samples used to assist with the estimation of δ. −1<p<1 is the offset between the assistant DTFT samples and the coarse frequency estimate $k_{m}\Delta f$. The meanings of Δf, δ, $k_{m}$, and *p*, and their relationships are also shown in Figure 1.

After some rearrangement, (5) can be simplified as

(6) $$X\left(k_{m}+p\right)=Ae^{j\phi }e^{j\pi \frac{N-1}{M}\left(\delta -p\right)}\frac{\sin(\pi (\delta -p))}{\sin(\frac{\pi }{M}\left(\delta -p\right))}.$$

## 3. Proposed Algorithm

To ensure the estimation accuracy of our model, we focus on the fine frequency estimation process and propose a new estimator. The steps of the proposed estimator are shown in Algorithm 1. First, we give the expressions of the two neighboring DTFT samples of the spectral peak:

(7) $$X\left(k_{m}\pm p\right)=Ae^{j\phi }e^{j2\pi \frac{N-1}{M}\left(\delta \mp p\right)}\frac{\sin(\frac{\pi N}{M}\left(\delta \mp p\right))}{\sin(\frac{\pi }{M}\left(\delta \mp p\right))}.$$

Then, we obtain their magnitudes:

(8) $$\left|X(k_{m}\pm p)\right|=A\left|\frac{\sin(\frac{\pi N}{M}\left(\delta \mp p\right))}{\sin(\frac{\pi }{M}\left(\delta \mp p\right))}\right|.$$

From the definitions of δ and *p*, it can be deduced that $\left|\delta \mp p\right|\;<3/2$. Considering that $0<\frac{N}{M}<1/2$, we can obtain $-3\pi /4<\frac{\pi N}{M}\left(\delta \mp p\right)<3\pi /4$. Thus, $\sin(\frac{\pi N}{M}\left(\delta \mp p\right))$ and $\sin(\frac{\pi }{M}\left(\delta \mp p\right))$ have the same sign in this range, namely, $\frac{\sin(\frac{\pi N}{M}\left(\delta \mp p\right))}{\sin(\frac{\pi }{M}\left(\delta \mp p\right))}>0$. In this way, (8) can be changed to

(9) $$\left|X(k_{m}\pm p)\right|=A\frac{\sin(\frac{\pi N}{M}\left(\delta \mp p\right))}{\sin(\frac{\pi }{M}\left(\delta \mp p\right))}.$$

The ratio of $\left|X(k_{m}+p)\right|$ to $\left|X(k_{m})\right|$ is

(10) $$\frac{\left|X(k_{m}+p)\right|}{\left|X(k_{m})\right|}=\frac{\sin(\frac{\pi N}{M}\left(\delta -p\right))}{\sin(\frac{\pi }{M}\left(\delta -p\right))}/\frac{\sin(\frac{\pi N}{M}\left(\delta \right))}{\sin(\frac{\pi }{M}\left(\delta \right))}.$$

Since M≫π(δ−p), we obtain

(11) $$\begin{array}{c} \frac{\left|X(k_{m}+p)\right|}{\left|X(k_{m})\right|}\approx \frac{\sin(\frac{\pi N}{M}\left(\delta -p\right))}{\sin(\frac{\pi N}{M}\left(\delta \right))}/\frac{\delta -p}{\delta }. \end{array}$$

(11) can also be expressed as:

(12) $$\frac{\left|X(k_{m}+p)\right|}{\left|X(k_{m})\right|}\frac{\delta -p}{\delta }\approx \frac{\sin(\frac{\pi N}{M}\left(\delta -p\right))}{\sin(\frac{\pi N}{M}\left(\delta \right))}.$$

According to the transformation formula of a trigonometric function, (12) can be decomposed as

(13) $$\begin{array}{c} \frac{\left|X(k_{m}+p)\right|}{\left|X(k_{m})\right|}\frac{\delta -p}{\delta }\approx \frac{\sin\left(\frac{\pi N}{M}\delta \right)\cos\left(\frac{\pi N}{M}p\right)-\cos\left(\frac{\pi N}{M}\delta \right)\sin\left(\frac{\pi N}{M}p\right)}{\sin(\frac{\pi N}{M}\delta )}. \end{array}$$

Similarly,

(14) $$\begin{array}{c} \frac{\left|X(k_{m}-p)\right|}{\left|X(k_{m})\right|}\frac{\delta +p}{\delta }\approx \frac{\sin\left(\frac{\pi N}{M}\delta \right)\cos\left(\frac{\pi N}{M}p\right)+\cos\left(\frac{\pi N}{M}\delta \right)\sin\left(\frac{\pi N}{M}p\right)}{\sin(\frac{\pi N}{M}\delta )}. \end{array}$$

After adding (13) to (14), we obtain

(15) $$\frac{\left|X(k_{m}+p)\right|}{\left|X(k_{m})\right|}\frac{\delta -p}{\delta }+\frac{\left|X(k_{m}-p)\right|}{\left|X(k_{m})\right|}\frac{\delta +p}{\delta }\approx 2\cos\left(\frac{\pi N}{M}p\right).$$

Finally, the estimate of δ can be obtained by

(16) $$\hat{\delta }=\frac{p\left|X(k_{m}+p)\right|-p\left|X(k_{m}-p)\right|}{\left|X(k_{m}+p)\right|+\left|X(k_{m}-p)\right|-2\left|X(k_{m})\right|\cos\left(\frac{\pi N}{M}p\right)}.$$

The frequency estimate is

(17) $$\hat{f}=\left(k_{m}+\hat{\delta }\right)\frac{f_{s}}{M}.$$

From (17), we can obtain a precise estimate of the frequency offset, but the above analysis is based on a signal without noise and some approximations. In fact, the frequency estimation derived from (17) usually deviates from the true frequency due to the influence of noise, so we introduce an iteration operation into the fine estimation process. The procedures of the proposed estimator are shown in Algorithm 1.
**Algorithm 1** The proposed iterative estimator.**Input:** Signal samples *y***Output:** Frequency estimate $\hat{f}$1:Coarse estimation: calculate the *M*-point DFT, and obtain the location of the spectral peak $k_{m}$ as the coarse frequency estimate.2:Fine estimation: initialize the iteration variables; $\hat{\delta }=0,q=1$3:**for** q<Q **do**4:      Compute the DTFT samples $\left|X(k_{m}+p)\right|$, $\left|X(k_{m})\right|$, and $\left|X(k_{m}-p)\right|$ according to (8)5:      Compute $\hat{\delta }$ according to (16)6:      Update the iteration variables: $k_{m}=k_{m}+\hat{\delta },q=q+1$7:Compute $\hat{f}$ according to (17)8:**return** $\hat{f}$

## 4. MSE Analysis of the Proposed Algorithm

To perform a MSE analysis for the developed iterative algorithm, we give the expression of the spectral magnitude of a complex exponential signal in noise:

(18) $$\begin{array}{c} \left|Y(k)\right|=\left|X(k)+W(k)\right|, \end{array}$$

where W(k) is the DFT of additive Gaussian white noise w(n). The spectral peak in the noise is

(19) $$\begin{array}{cc} \left|Y(k_{m})\right|\;= & \left|X\left(k_{m}\right)+W\left(k_{m}\right)\right|=\left|A\frac{\sin\left(\frac{\pi N}{M}\delta \right)}{\sin\left(\frac{\pi }{M}\delta \right)}+W\left(k_{m}\right)e^{-j\phi }e^{-j\pi \frac{N-1}{M}\delta }\right|. \end{array}$$

Because $\left|W(k)\right|\ll \left|X(k)\right|$ at a high *SNR*, $|Y_{k_{m}}|=A_{k_{m}}+Re(e^{-j\varphi_{k_{m}}}W_{k_{m}}$), $A_{k_{m}}$ is the magnitude of $X_{k_{m}}$, and $\varphi_{k_{m}}$ is the phase of $X_{k_{m}}$. Therefore, an approximation of (19) can be expressed as

(20) Y(km)≈X(km)+Re(W(km)e−jφkm)≈Akm+Ukm,

where $A_{k_{m}}=\;\left|X(k_{m})\right|=A\cdot \sin\left(\frac{\pi N}{M}\delta \right)/\sin\left(\frac{\pi }{M}\delta \right)$, $U_{k_{m}}=Re\left(W\left(k_{m}\right)e^{-j\varphi_{k_{m}}}\right)$, $\varphi_{k_{m}}=\phi +\pi \frac{N-1}{M}\delta$. Similarly, the DTFT samples can be expressed as $\left|Y(k_{m}+p)\right|\approx A_{k_{m}+p}+U_{k_{m}+p}$, $\left|Y(k_{m}-p)\right|\approx A_{k_{m}-p}+U_{k_{m}-p}$. To simplify the analysis, we denote $Y_{0}=Y\left(k_{m}\right)$, $Y_{p}=Y\left(k_{m}+p\right)$, $Y_{-p}=Y\left(k_{m}-p\right)$, $A_{0}=A_{k_{m}}$, $A_{p}=A_{k_{m}+p}$, $A_{-p}=A_{k_{m}-p}$, $U_{0}=U_{k_{m}}$, $U_{p}=U_{k_{m}+p}$, and $U_{-p}=U_{k_{m}-p}$. Consequently, (16) becomes

(21) $$\begin{array}{cc} \hat{\delta } & =\frac{p\left(A_{p}+U_{p}\right)-p\left(A_{-p}+U_{-p}\right)}{\left(A_{p}+U_{p}\right)+\left(A_{-p}+U_{-p}\right)-2\left(A_{0}+U_{0}\right)\cos\left(\frac{\pi N}{M}p\right)}\\ \; & =\frac{p(A_{p}-A_{-p}+U_{p}-U_{-p})}{A_{p}+A_{-p}-2A_{0}\cos\left(\frac{\pi N}{M}p\right)+U_{p}+U_{-p}-2U_{0}\cos\left(\frac{\pi N}{M}p\right)}\\ \; & =\frac{p(A_{p}-A_{-p}+U_{p}-U_{-p})}{A_{p}+A_{-p}-2A_{0}\cos\left(\frac{\pi N}{M}p\right)}\cdot \frac{1}{1+\frac{U_{p}+U_{-p}-2U_{0}\cos\left(\frac{\pi N}{M}p\right)}{A_{p}+A_{-p}-2A_{0}\cos\left(\frac{\pi N}{M}p\right)}}. \end{array}$$

Because $\frac{U_{p}+U_{-p}-2U_{0}\cos\left(\frac{\pi N}{M}p\right)}{A_{p}+A_{-p}-2A_{0}\cos\left(\frac{\pi N}{M}p\right)}\ll 1$, the first-order Taylor expansion of the second item on the right side of (21) is

(22) $$\frac{1}{1+\frac{U_{p}+U_{-p}-2U_{0}\cos\left(\frac{\pi N}{M}p\right)}{A_{p}+A_{-p}-2A_{0}\cos\left(\frac{\pi N}{M}p\right)}}\approx 1-\frac{U_{p}+U_{-p}-2U_{0}\cos\left(\frac{\pi N}{M}p\right)}{A_{p}+A_{-p}-2A_{0}\cos\left(\frac{\pi N}{M}p\right)}.$$

By introducing (22) into (21), the frequency offset is

(23) $$\begin{array}{cc} \hat{\delta }= & \frac{p(A_{p}-A_{-p})}{A_{p}+A_{-p}-2A_{0}\cos\left(\frac{\pi N}{M}p\right)}+\frac{p(U_{p}-U_{-p})}{A_{p}+A_{-p}-2A_{0}\cos\left(\frac{\pi N}{M}p\right)}\\ \; & -\frac{p\left(A_{p}-A_{-p}\right)\left(U_{p}+U_{-p}-2U_{0}\cos\left(\frac{\pi N}{M}p\right)\right)}{{\left(A_{p}+A_{-p}-2A_{0}\cos\left(\frac{\pi N}{M}p\right)\right)}^{2}}\\ \; & -\frac{p\left(U_{p}-U_{-p}\right)\left(U_{p}+U_{-p}-2U_{0}\cos\left(\frac{\pi N}{M}p\right)\right)}{{\left(A_{p}+A_{-p}-2A_{0}\cos\left(\frac{\pi N}{M}p\right)\right)}^{2}}. \end{array}$$

The last item is approximately zero if the *SNR* is high. Thus, after some simplification, (23) becomes

(24) $$\begin{array}{cc} \hat{\delta } & =\delta +\frac{p(U_{p}-U_{-p})}{A_{p}+A_{-p}-2A_{0}\cos\left(\frac{\pi N}{M}p\right)}-\delta \cdot \frac{U_{p}+U_{-p}-2U_{0}\cos\left(\frac{\pi N}{M}p\right)}{A_{p}+A_{-p}-2A_{0}\cos\left(\frac{\pi N}{M}p\right)}. \end{array}$$

Then, we compute the expectation of the estimation error and the MSE of the frequency estimate:

(25) $$\begin{array}{cc} E(\hat{\delta }-\delta ) & =\frac{p(U_{p}-U_{-p})}{A_{p}+A_{-p}-2A_{0}\cos\left(\frac{\pi N}{M}p\right)}-\delta \cdot \frac{U_{p}+U_{-p}-2U_{0}\cos\left(\frac{\pi N}{M}p\right)}{A_{p}+A_{-p}-2A_{0}\cos\left(\frac{\pi N}{M}p\right)}\\ \; & =\frac{\left(p-\delta \right)U_{p}-\left(p+\delta \right)U_{-p})-2U_{0}\delta \cos\left(\frac{\pi N}{M}p\right)}{A_{p}+A_{-p}-2A_{0}\cos\left(\frac{\pi N}{M}p\right)}, \end{array}$$

and

(26) $$\begin{array}{c} E\left[{\left(\hat{\delta }-\delta \right)}^{2}\right]=E\left[\frac{\left(p-\delta \right)U_{p}-\left(p+\delta \right)U_{-p})}{A_{p}+A_{-p}-2A_{0}\cos\left(\frac{\pi N}{M}p\right)}\right.{\left.-\frac{2U_{0}\delta \cos\left(\frac{\pi N}{M}p\right)}{A_{p}+A_{-p}-2A_{0}\cos\left(\frac{\pi N}{M}p\right)}\right]}^{2}. \end{array}$$

According to deduction in Appendix A, the final MSE expression is obtained as follows:

(27) $$\begin{array}{c} E\left[{\left(\hat{\delta }-\delta \right)}^{2}\right]\approx \left\{\begin{array}{c} \begin{array}{c} \frac{\pi^{2}N}{M^{2}}\cdot \frac{\delta^{2}{\left(\left(\delta^{2}-p^{2}\right)\right)}^{2}}{SNR}\cdot \\ \frac{p^{2}+\delta^{2}+2\delta^{2}\cos^{2}\left(\frac{\pi N}{M}p\right)-\left(3\delta^{2}+p^{2}\right)\sin c\left(\frac{2\pi N}{M}p\right)}{4p^{2}{\left(p\sin\left(\frac{\pi N}{M}\delta \right)\cos\left(\frac{\pi N}{M}p\right)-\delta \cos\left(\frac{\pi N}{M}\delta \right)\sin\left(\frac{\pi N}{M}p\right)\right)}^{2}},\\ \left|\delta \right|\neq p\&\left|\delta \right|\neq 0 \end{array}\\ \frac{p^{2}}{4N\cdot SNR}\cdot \frac{1-\sin c\frac{2\pi N}{M}p}{{\left(\sin c\frac{\pi N}{M}p-\cos\left(\frac{\pi N}{M}p\right)\right)}^{2}},\delta =0\\ \frac{2p^{2}}{N\cdot SNR}\cdot \frac{1+\cos^{2}\left(\frac{\pi N}{M}p\right)-2\sin c\frac{2\pi N}{M}p}{{\left(1-\sin c\frac{2\pi N}{M}p\right)}^{2}},\left|\delta \right|=p \end{array}\right. \end{array}.$$

### 4.1. Discussion on *p*

Once the ratio M/N has been decided, a smaller *p* means that the assistant DTFT samples are closer to the DFT peak. Will we obtain a more precise estimate if a smaller *p* is chosen? How can we choose *p*? This section provides a discussion about *p*. We simulate the proposed method versus *p* for different values of δ to verify the influence of *p* on the estimation accuracy of the resulting model.

**Figure 2:**
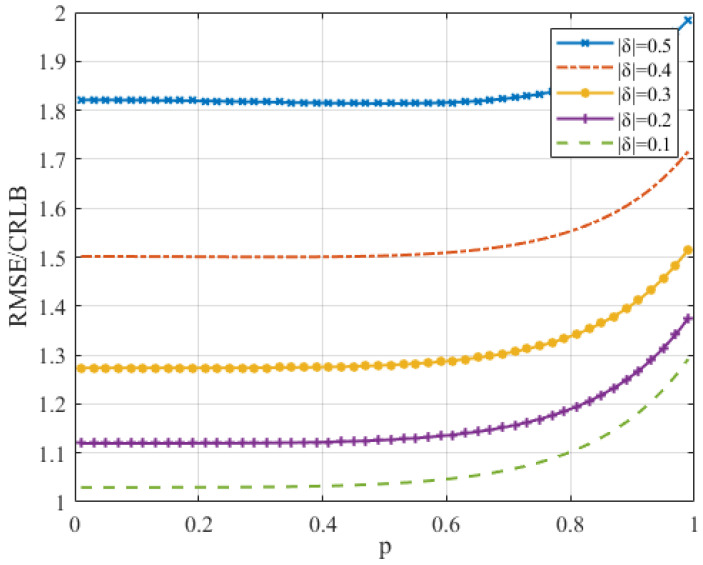
The RMSE/CRLB of the proposed algorithm versus *p* for different values of δ when the SNR=0 dB and N=512.

In Figure 2, *p* has an important influence on the RMSE/CRLB of the algorithm. It decreases as *p* decreases at the beginning. However, when *p* is sufficiently small, the improvement in the RMSE/CRLB does not change further. In fact, it does not keep decreasing as *p* decreases. The minimum RMSE/CRLB varies with $\left|\delta \right|$, so we choose a medium p=0.3 in Section 5.

### 4.2. Discussion on *M*

**Figure 3:**
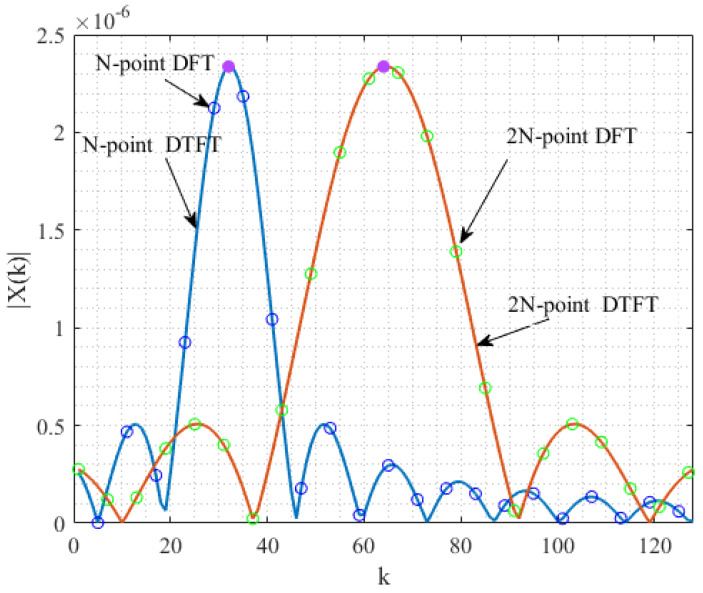
DFTs and DTFTs of complex exponential signals at M=N and M=2N.

**Figure 4:**
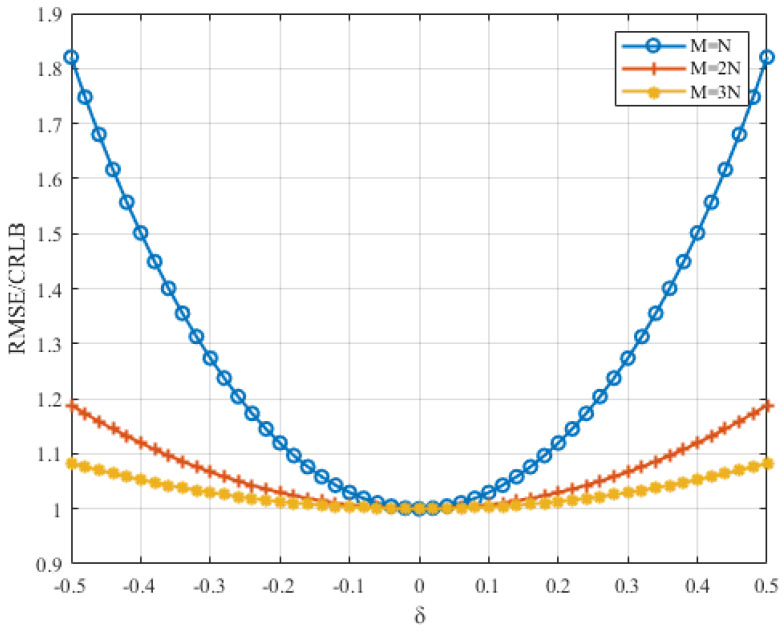
The RMSE/CRLB of the proposed estimator versus δ at different values of *M* when SNR=0 dB and N=512.

The spectral frequency resolution directly affects the algorithm’s estimation accuracy. When the sampling frequency is determined, choosing a larger *N* can reduce the spectral frequency resolution and increase the spectral magnitude. However, this also increases the estimation time and computational complexity of the algorithm. Therefore, we first pad zeros after the signal samples before computing the corresponding DFTs. This approach can decrease the frequency resolution without increasing the observation time. The DFT and DTFT waveforms of samples with and without zero-padding are shown in Figure 3. The figure indicates that the DFT and DTFT waveforms of the signal are extended after the signal samples are padded with *N* zeros. The two green circles are closer to the peak than the two blue circles. The 2N-point DFT around the peak, which is closer to the peak, can obtain a smaller RMSE/CRLB in Figure 4. In addition, as M/N increases, the improvement in the MSE decreases. Considering the computational complexity brought by *M*, we choose M=2N as a compromise between computational complexity and the estimation accuracy.

### 4.3. Discussion on *Q*

**Figure 5:**
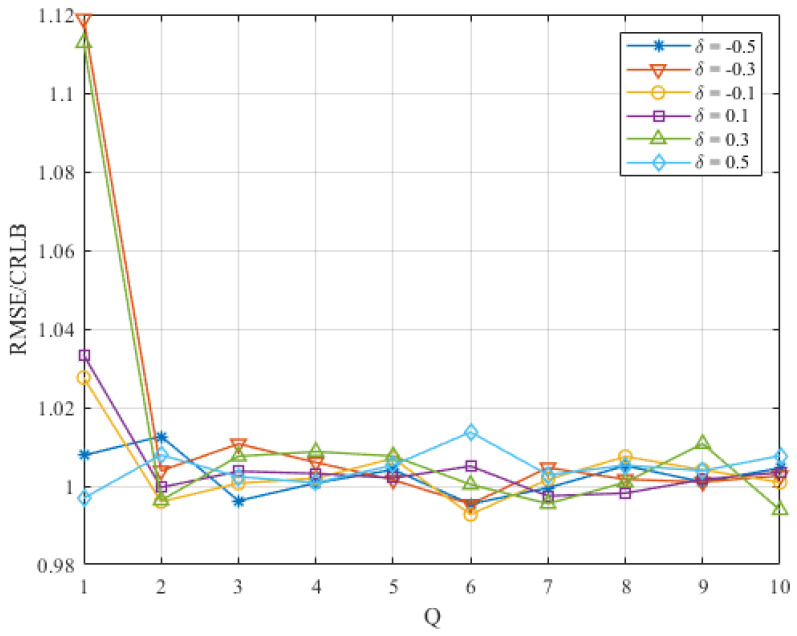
The RMSE/CRLB of the proposed estimator versus *Q* at different values of δ when SNR=0 dB and N=512.

We introduce the iteration operation into the frequency estimation and decrease the final estimation error by iteratively updating the estimate of the frequency offset and the DTFT samples used in interpolation. To choose the number of iteration, we make a simulation about the performance of the proposed algorithm versus iteration number *Q* at different δ when SNR=0 dB and N=512. The RMSE/CRLB is shown in Figure 5. From Figure 5, it can be seen that the RMSE/CRLB decreases obviously after the second iteration, when $\left|\delta \right|=0.5$ or $\left|\delta \right|=0.3$. However, When Q>2, the RMSE/CRLB does not decrease as Q increases. Thus, considering the accuracy improvement and the calculation complexity, we choose Q=2 as the iteration number for simulation in Section 5. This iteration number is also consistent with the research in [24].

## 5. Experiment

To test the estimation performance of the proposed iterative estimator, we set the simulation parameters as follows: the signal magnitude A=1, the sampling frequency $f_{s}=512$ kHz, the frequency offset $\left|\delta \right|<0.5$, the spectral peak $k_{m}$ = 64 kHz, the true frequency 63.5 kHz < $f_{0}$ < 64.5 kHz, the number of iterations Q=2 [24], and the initial phase ϕ∈[0,2π] is generated randomly. Moreover, we compare the proposed algorithm with other algorithms including Jacobsen estimator [7], Candan estimator [8,9], A&M estimator [16], Fan estimator [15], and Fang estimator [13], and RCSTL [14]. Every estimation result is obtained from 10,000 simulations.

### 5.1. Comparison of the CRLB and RMSE of the Proposed Estimator

**Figure 6:**
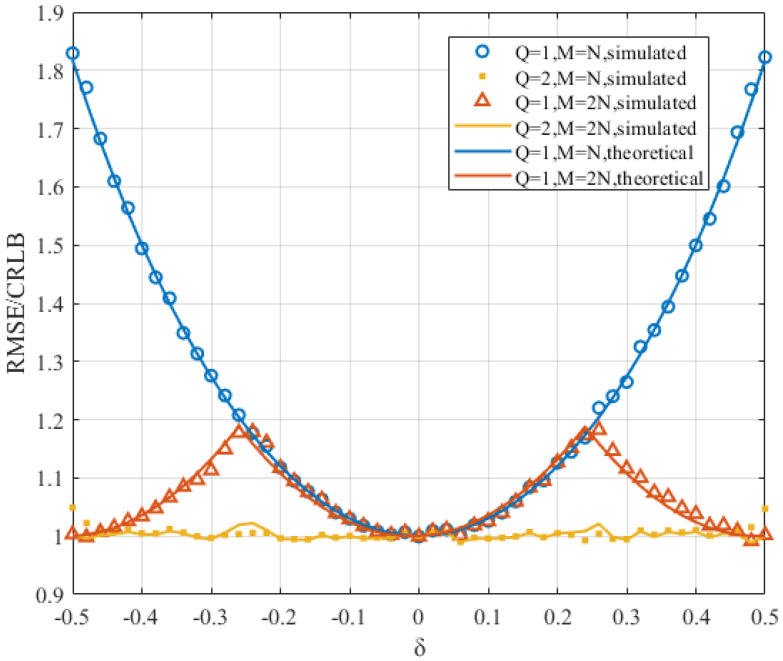
The RMSE/CRLB of the proposed estimator versus δ under different settings of *M* and *Q* when SNR=0 dB and N=512.

In this section, we perform simulations of the proposed estimator with different iterations. As shown in Figure 6, the simulation result at the first iteration is consistent with the MSE analysis. The periodic characteristic is also verified. Therefore, the MSE analysis in Section 4 is effective. However, these results provide only the reference standard for the proposed estimator at the first iteration. The RMSE/CRLB at the second iteration can be verified by simulation. In Figure 6, the RMSE/CRLB at the second iteration can approach 1, which means that the proposed estimator has a promising estimation accuracy.

One more aspect that needs to be explained is that the MSE of the proposed estimator in (27) is a periodic function. It can be seen in Figure 6 that between δ=−0.5 and δ=0.5, there are two periods for M=2N and Q=1 and one period for M=N and Q=1. They are both symmetric about δ=0. This means that the RMSE/CLRB is periodical and that the length of its period varies with the ratio of *M* to *N*.

### 5.2. Comparison of RMSEs among the Existing Algorithms

To test the proposed estimator’s RMSE versus the *SNR* and *N*, we set δ=0.2 and simulate seven estimators.

**Figure 7:**
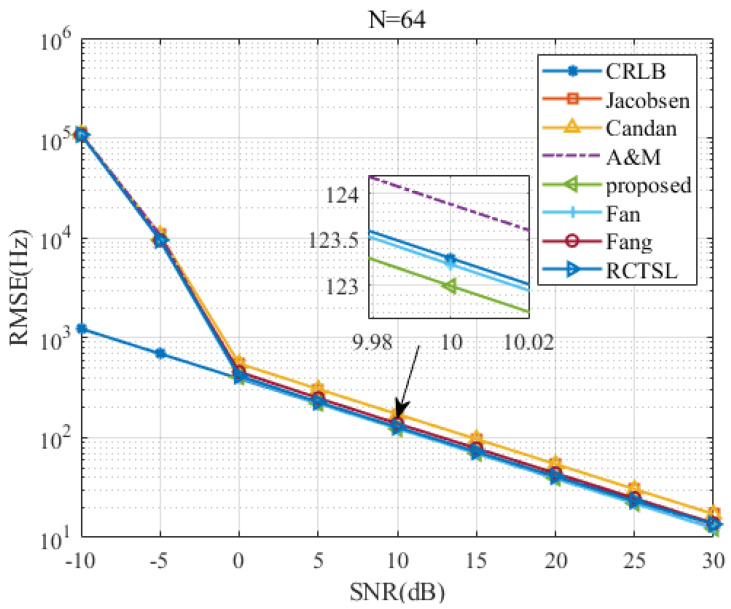
RMSE comparison among frequency estimates versus *SNR* at N=64 and δ=0.2.

**Figure 8:**
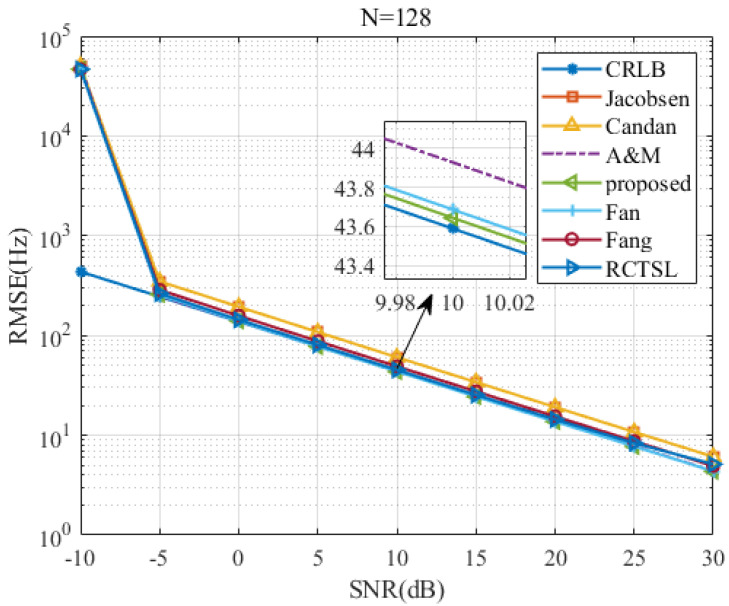
RMSE comparison among frequency estimates versus *SNR* at N=128 and δ=0.2.

**Figure 9:**
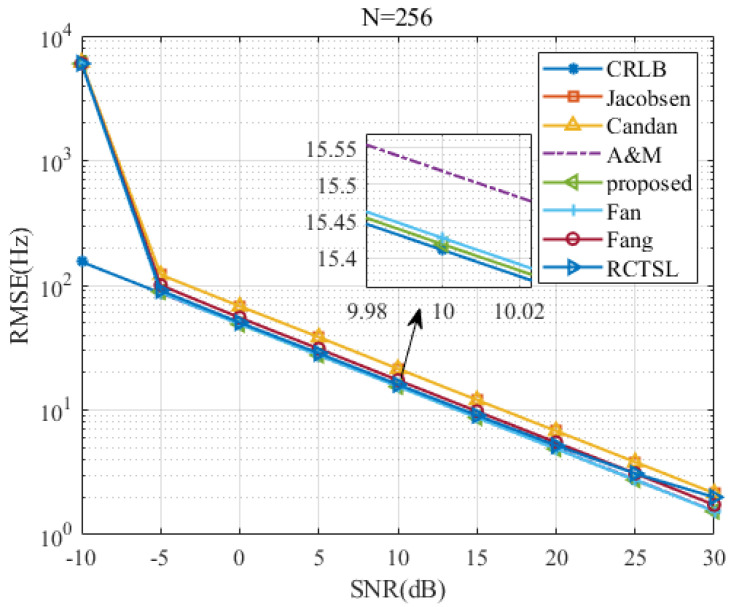
RMSE comparison among frequency estimates versus *SNR* at N=256 and δ=0.2.

**Figure 10:**
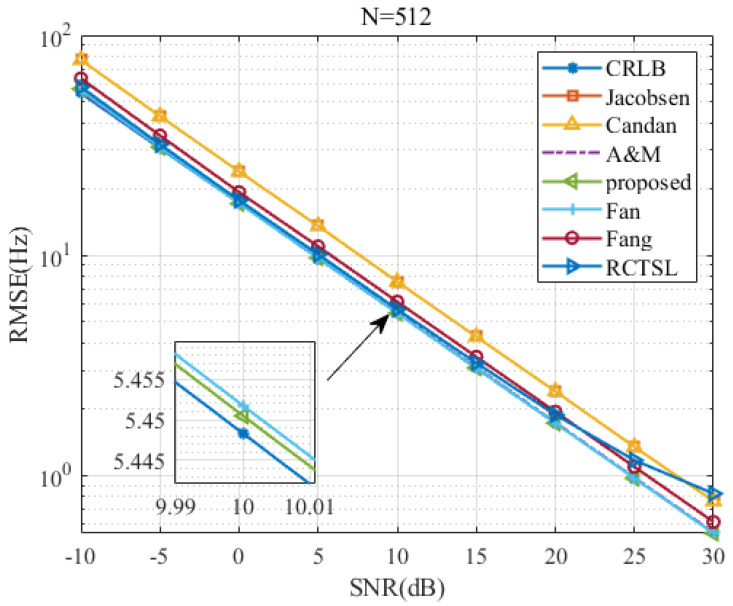
RMSE comparison among frequency estimates versus *SNR* at N=512 and δ=0.2.

Figure 7, Figure 8, Figure 9 and Figure 10 are figures corresponding to N=64, N=128, N=256, and N=512, respectively. Upon comparing these figures, the *SNR* threshold decreases from 0 dB to −10 dB. The RMSEs of these estimators and the RMSE differences among these estimators decrease as *N* increases. Detailed figures at SNR=10 dB are also provided, from which we can see that the RMSEs of A&M, Fan, and the proposed estimator are closest to the CRLB. The RMSE of the proposed estimator is slightly lower than those of the other simulated methods in most cases, although the RMSE difference between the proposed estimator and the Fan estimator is small. In addition, the RMSE of the proposed estimator is only 1.003 times the CRLB when SNR=10 dB and N=512.

### 5.3. RMSE Comparison among Estimators with a Comparable Accuracy

**Figure 11:**
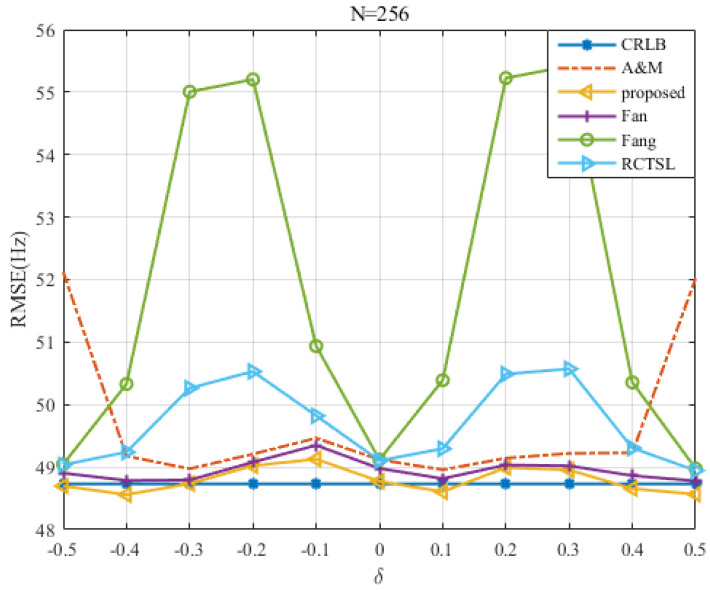
RMSE comparison among the frequency estimates versus δ at N=256 and SNR=0 dB.

**Figure 12:**
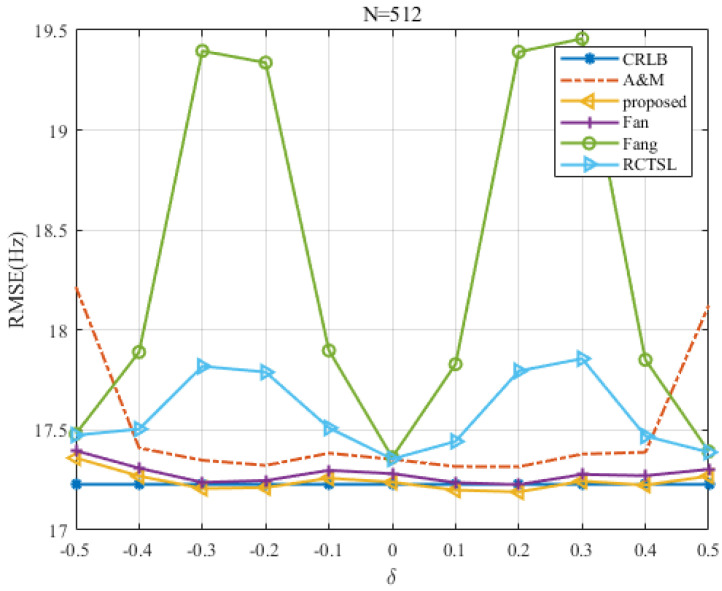
RMSE comparison among the frequency estimates versus δ at N=512 and SNR=0 dB.

To further evaluate the RMSE of the proposed estimator, we compare the estimators with the comparable estimation accuracy at SNR=0 dB. The results in Figure 11 and Figure 12 show that the RMSE of the proposed estimator is closest to the CRLB in most cases. RMSE of the Fan estimator is slightly larger than that of the proposed estimator. Both the two estimators are more stable than the other simulated estimators, showing that the variations in their RMSEs are small throughout the whole δ range. The RMSE of the A&M estimator is also very small, but there exists a kind of edge effect in which the RMSE increases at δ=±0.5. For some values of δ, the corresponding RMSEs may lie slightly below the CRLB and are asymmetric relative to δ=0 due to the limited number of Monte Carlo trials.

### 5.4. Computational Complexities of the Estimators Used in the Simulation

Computational complexity is an important aspect to consider when evaluating the performance of estimators. We mainly compare the numbers of complex calculations performed by the various methods, including complex multiplications and complex additions.

Table 2 gives the computational complexities of all simulated estimators. To reduce the computational complexity of the DFT, we choose $N=2^{k}$ as the DFT length and compute the FFT instead. An *N*-point FFT requires $\frac{N}{2}log_{2}N$ complex multiplications and $Nlog_{2}N$ complex additions. A one-bin *N*-point DTFT requires *N* complex multiplications and N−1 complex additions. The additional multiplication and addition operation other than those utilized in the FFT calculation are performed to compute $\hat{\delta }$. During the two iterations, the proposed estimator uses five magnitudes of DTFTs to compute $\hat{\delta }$, so it omits several complex multiplications and additions compared with the Fan estimator, which have comparable estimation accuracies as the proposed estimator. Compared with the A&M estimator, the proposed estimator has a lower RMSE and avoids the edge effect when $\left|\delta \right|$ approaches to 0.5.

## 6. Conclusions

In this paper, we present a new and simple iterative frequency estimator that is used for the fine estimation of complex exponential signals. In this algorithm, the magnitudes of DTFT samples are used to calculate the frequency offset to reduce the number of required complex calculations, and iterative calculations are introduced into the estimation process to further improve the estimation accuracy of the resulting model. It is verified by simulation that the developed algorithm achieves a higher estimation accuracy compared with other algorithms. Moreover, it has a lower computational complexity than the algorithm that has a comparable estimation accuracy. In addition, the general form of the proposed estimator is also presented in this paper to help select appropriate parameters according to given application requirements.

## Figures and Tables

**Table 1:** Analysis of key estimators.

Estimators	Merits/Demerits	Limits of Applicability
IpDFT with a cosine window [20]	Reduce the influence of spectral leakage by introducing a cosine window/increase the computation	Linear computation based on all DFT samples
IpDTFT with a cosine window [21]	Reduce the influence of spectral leakage by introducing a cosine window/increase the computation, extra two DTFT computations	Almost two times CRLB
e-FLLs/FLLs (frequency-domain linear least-squares) [22,23]	Robust to harmonics/matrix computation for window, matrix inversion for linear least-squares	Amplitude and phase estimation
QSE/HAQSE (hybrid A&M and q-shift estimator) [24]	DFT-based, easily realizable, within ±0.003 dB CRLB/at least extra four DTFT computations and several complex computations for the estimate	Edge effect
Aboutanios and Mulgrew (A&M) [25,26]	DFT-based, easily realizable, 1.0147 times the asymptotic CRLB/extra four DTFT computations and several complex computations for the estimate	Edge effect
Parabolic interpolation [27]	DFT-based, easily realizable, within ±0.526 dB CRLB/extra three DTFT computations and several complex computations for the estimate	Limited accuracy

**Table 2:** Computational complexities of the estimators used in the simulation.

Estimators	Complex Multiplications	Complex Additions
Jacobsen (*N* points)	$\frac{N}{2}log_{2}N+1$	$Nlog_{2}N+3$
Candan (*N* points)	$\frac{N}{2}log_{2}N+1$	$Nlog_{2}N+3$
A&M (*N* points, 2 iterations)	$\frac{N}{2}log_{2}N+4N+2$	$Nlog_{2}N+4N$
Fan (*M* points, 2 iterations)	$\frac{M}{2}log_{2}M+5N+8$	$Mlog_{2}M+5N+1$
Fang (*M* points)	$\frac{M}{2}log_{2}M$	$Mlog_{2}M$
RCSTL (*M* points)	$\frac{M}{2}log_{2}M+1$	$Mlog_{2}M$
Proposed (*M* points, 2 iterations)	$\frac{M}{2}log_{2}M+5N$	$Mlog_{2}M+5\left(N-1\right)$

## Data Availability

Data available on request due to restrictions. The data presented in this study are available on request from the corresponding author. The data are not publicly available due to privacy.

## Appendix A. Deduction of the MSE Expression

The deduction process of the final expression of the MSE is given in this section. To obtain $E\left[{\left(\hat{\delta }-\delta \right)}^{2}\right]$, we need to compute the numerator and denominator of the MSE:

(A1) $$\begin{array}{cc} {{\left(\hat{\delta }-\delta \right)}^{2}}_{nomi}= & {\left(p-\delta \right)}^{2}U_{p}^{2}+{\left(p+\delta \right)}^{2}U_{-p}^{2}+4U_{0}^{2}\delta^{2}\cos^{2}\left(\frac{\pi N}{M}p\right)\\ \; & -2\left(p^{2}-\delta^{2}\right)U_{p}U_{-p}+4U_{p}U_{+p}\left(p-\delta \right)\delta \cos\left(\frac{\pi N}{M}p\right)\\ \; & +4U_{p}U_{-p}\left(p+\delta \right)\delta \cos\left(\frac{\pi N}{M}p\right), \end{array}$$

and

(A2) $${{\left(\hat{\delta }-\delta \right)}^{2}}_{\mathrm{de}nomi}={\left(A_{p}+A_{-p}-2A_{0}\cos\left(\frac{\pi N}{M}p\right)\right)}^{2}.$$

Because $U_{p}$ is the real part of $W_{p}$ at all frequencies and $w\left(n\right)∼N\left(0,\sigma^{2}\right)$, $E\left(U_{p}^{2}\right)=E\left(U_{-p}^{2}\right)=E\left(U_{0}^{2}\right)=N\sigma^{2}/2$. To compute $E(U_{p}U_{+p})$, we go back to the DTFT expression:

(A3) Up=Re(Wpe−jφp)=Re(∑n=0N−1w(n)e−j(2πfpn+φp)).

The expectation of $U_{p}U_{-p}$ is

(A4) $$\begin{array}{cc} E\left[U_{p}U_{-p}\right] & =E\left[\sum_{n=0}^{N-1}\left(w_{r}\left(n\right)\cos\left(2\pi f_{p}n+\varphi_{p}\right)+\left.w_{i}\left(n\right)\sin\left(2\pi f_{p}n+\varphi_{p}\right)\right)\cdot \right.\right.\\ \; & \left.\sum_{m=0}^{N-1}\left(w_{r}\left(m\right)\cos\left(2\pi f_{-p}m+\varphi_{-p}\right)+\left.w_{i}\left(m\right)\sin\left(2\pi f_{-p}m+\varphi_{-p}\right)\right)\right.\right], \end{array}$$

where $\varphi_{p}=\varphi +\pi \frac{N-1}{M}\left(\delta -p\right)$ and $\varphi_{-p}=\varphi +\pi \frac{N-1}{M}\left(\delta +p\right)$ are the phases of $X_{p}$ and $X_{-p}$, respectively. $f_{p}=\frac{N}{M}\left(\delta -p\right)$ and $f_{-p}=\frac{N}{M}\left(\delta +p\right)$ are the frequencies of $X_{p}$ and $X_{-p}$, respectively. According to the independence characteristic of Gaussian white noise, $E(w_{r}\left(n\right)w_{i}\left(m\right))=0$, and $E(w_{i}\left(n\right)w_{r}\left(m\right))=0$. If n≠m, $E(w_{r}\left(n\right)w_{r}\left(m\right))=0$, and $E(w_{i}\left(n\right)w_{i}\left(m\right))=0$. Therefore, only autocorrelation items (n=m) are retained in the expectation, which can be written as

(A5) $$\begin{array}{cc} E\left[U_{p}U_{-p}\right]= & E[\sum_{n=0}^{N-1}\left(w_{r}^{2}\left(n\right)\cos\left(2\pi f_{p}n+\varphi_{p}\right)\cos\left(2\pi f_{-p}n+\varphi_{-p}\right)+\right.\\ \; & \left.w_{i}^{2}\left(n\right)\sin\left(2\pi f_{p}n+\varphi_{p}\right)\sin\left(2\pi f_{-p}n+\varphi_{-p}\right)\right)]\\ = & N\sigma^{2}/2\sum_{n=0}^{N-1}\left(\cos\left(2\pi f_{p}n+\varphi_{p}\right)\cos\left(2\pi f_{-p}n+\varphi_{-p}\right)\right.\\ \; & \left.+\sin\left(2\pi f_{p}n+\varphi_{p}\right)\sin\left(2\pi f_{-p}n+\varphi_{-p}\right)\right)\\ = & N\sigma^{2}/2\sum_{n=0}^{N-1}\left(\cos(2\pi \left(f_{p}-f_{-p}\right)n+\left(\varphi_{p}-\varphi_{-p}\right))\right). \end{array}$$

After some algebra, we have

(A6) $$\begin{array}{cc} E\left[U_{p}U_{-p}\right]= & \frac{N\sigma^{2}}{4}\left[e^{j(\varphi_{p}-\varphi_{-p})}\frac{1-e^{j(2\pi \left(f_{p}-f_{-p}\right)n)}}{1-e^{j(2\pi \left(f_{p}-f_{-p}\right)n/M)}}+\right.\\ \; & \left.e^{-j(\varphi_{p}-\varphi_{-p})}\frac{1-e^{-j(2\pi \left(f_{p}-f_{-p}\right)n)}}{1-e^{-j(2\pi \left(f_{p}-f_{-p}\right)n/M)}}\right]. \end{array}$$

By substituting $\varphi_{p}-\varphi_{-p}=-\pi \frac{N-1}{M}p,f_{p}-f_{-p}=-\frac{2N}{M}p$ into (A6), we obtain

(A7) $$E\left[U_{p}U_{-p}\right]=\frac{N\sigma^{2}}{2}\frac{\sin(\frac{2\pi Np}{M})}{\sin(\frac{2\pi p}{M})}\approx \frac{N\sigma^{2}}{2}\frac{\sin(\frac{2\pi Np}{M})}{\frac{2\pi p}{M}}.$$

In the same way, $E\left[U_{0}U_{p}\right]=E\left[U_{0}U_{-p}\right]=N\sigma^{2}/2\frac{\sin(\pi Np/M)}{\sin(\pi p/M)}\approx N\sigma^{2}/2\frac{\sin(\pi Np/M)}{\pi p/M}$. Then, we introduce the above results into (A1) and obtain

(A8) $$\begin{array}{c} E{\left[{\left(\hat{\delta }-\delta \right)}^{2}\right]}_{nomi}=N\sigma^{2}\left[p^{2}+\delta^{2}+2\delta^{2}\cos^{2}\left(\frac{\pi N}{M}p\right)-\right.\left.\left(3\delta^{2}+p^{2}\right)\frac{\sin\left(\frac{2\pi N}{M}p\right)}{\frac{2\pi }{M}p}\right]. \end{array}$$

To compute the denominator of $E\left[{\left(\hat{\delta }-\delta \right)}^{2}\right]$, we return to the expression of $A_{p}$

(A9) $$\begin{array}{cc} A_{p}=\left|X(k_{m}+p)\right| & =A\cdot \frac{\sin\left(\frac{\pi N}{M}\left(\delta -p\right)\right)}{\sin\left(\frac{\pi }{M}\left(\delta -p\right)\right)}\approx A\cdot \frac{\sin\left(\frac{\pi N}{M}\left(\delta -p\right)\right)}{\frac{\pi }{M}\left(\delta -p\right)}. \end{array}$$

Similarly, $A_{-p}\approx A\cdot \frac{\sin\left(\frac{\pi N}{M}\left(\delta +p\right)\right)}{\frac{\pi }{M}\left(\delta +p\right)}$, $A_{0}\approx A\cdot \frac{\sin\left(\frac{\pi N}{M}\delta \right)}{\frac{\pi }{M}\delta }$.

By introducing $A_{p}$, $A_{-p}$, and $A_{0}$ into (A2), we obtain

(A10) $$\begin{array}{cc} A_{p}+A_{-p}-2A_{0}\cos\left(\frac{\pi N}{M}p\right)\approx & A\cdot \left[\frac{\sin\left(\frac{\pi N}{M}\left(\delta -p\right)\right)}{\frac{\pi }{M}\left(\delta -p\right)}+\frac{\sin\left(\frac{\pi N}{M}\left(\delta +p\right)\right)}{\frac{\pi }{M}\left(\delta +p\right)}\right.\\ \; & -\left.\frac{2\sin\left(\frac{\pi N}{M}\delta \right)\cos\left(\frac{\pi N}{M}p\right)}{\frac{\pi }{M}\delta }\right]\\ \approx & \frac{2AM}{\pi }\cdot \left(\frac{p^{2}\sin\left(\frac{\pi N}{M}\delta \right)\cos\left(\frac{\pi N}{M}p\right)}{\delta (\delta^{2}-p^{2})}\right.\\ \; & -\left.\frac{p\delta \cos\left(\frac{\pi N}{M}\delta \right)\sin\left(\frac{\pi N}{M}p\right)}{\delta (\delta^{2}-p^{2})}\right). \end{array}$$

Then,

(A11) $$\begin{array}{cc} E{\left[{\left(\hat{\delta }-\delta \right)}^{2}\right]}_{\mathrm{de}nomi}= & {\left(A_{p}+A_{-p}-2A_{0}\cos\left(\frac{\pi N}{M}p\right)\right)}^{2}\\ = & {\left(\frac{2AMp}{\pi }\right)}^{2}\cdot \frac{\left[p\sin\left(\frac{\pi N}{M}\delta \right)\cos\left(\frac{\pi N}{M}p\right)\right.}{\delta^{2}{\left(\delta^{2}-p^{2}\right)}^{2}}\\ \; & -\frac{{\left.\delta \cos\left(\frac{\pi N}{M}\delta \right)\sin\left(\frac{\pi N}{M}p\right)\right]}^{2}}{\delta^{2}{\left(\delta^{2}-p^{2}\right)}^{2}}. \end{array}$$

Since there are two cases in which the denominator equals zero, which leads to a meaningless result, we further analyze these two cases. When $\left|\delta \right|=p$,

(A12) $$\begin{array}{cc} \; & A_{p}+A_{-p}-2A_{0}\cos\left(\frac{\pi N}{M}p\right)\\ \; & \approx \frac{2AMp}{\pi }\cdot \frac{p\sin\left(\frac{\pi N}{M}\delta \right)\cos\left(\frac{\pi N}{M}p\right)-\delta \cos\left(\frac{\pi N}{M}\delta \right)\sin\left(\frac{\pi N}{M}p\right)}{\delta (\delta^{2}-p^{2})}\\ \; & \approx \frac{2AMp}{\pi }\cdot \frac{\delta \sin\left(\frac{\pi N}{M}\left(\delta -p\right)\right)-\left(\delta -p\right)\sin\left(\frac{\pi N}{M}\delta \right)\cos\left(\frac{\pi N}{M}p\right)}{\delta (\delta^{2}-p^{2})}\\ \; & \approx \frac{2ANp}{\delta +p}\cdot \left(\sin c\left(\frac{\pi N}{M}\left(\delta -p\right)\right)-\sin c\left(\frac{\pi N}{M}\delta \right)\cos\left(\frac{\pi N}{M}p\right)\right)\\ \; & \approx AN\cdot \left(1-\sin c\left(\frac{\pi N}{M}\delta \right)\cos\left(\frac{\pi N}{M}p\right)\right); \end{array}$$

when δ=0,

(A13) $$\begin{array}{cc} \; & A_{p}+A_{-p}-2A_{0}\cos\left(\frac{\pi N}{M}p\right)\\ \; & \approx \frac{2AM}{\pi }\cdot \frac{p^{2}\sin\left(\frac{\pi N}{M}\delta \right)\cos\left(\frac{\pi N}{M}p\right)-p\delta \cos\left(\frac{\pi N}{M}\delta \right)\sin\left(\frac{\pi N}{M}p\right)}{\delta (\delta^{2}-p^{2})}\\ \; & \approx \frac{2AM}{\pi }\cdot \left(-\frac{\sin\left(\frac{\pi N}{M}\delta \right)\cos\left(\frac{\pi N}{M}p\right)}{\delta }+\frac{\cos\left(\frac{\pi N}{M}\delta \right)\sin\left(\frac{\pi N}{M}p\right)}{p}\right)\\ \; & \approx 2AN\cdot \left(\sin c\left(\frac{\pi N}{M}p\right)-\cos\left(\frac{\pi N}{M}p\right)\right). \end{array}$$
